# Seminal plasma protein profiles and testosterone levels as biomarker semen quality of candidate Madura bulls

**DOI:** 10.5455/javar.2023.j696

**Published:** 2023-09-24

**Authors:** Nurul Azizah, Suherni Susilowati, Budi Utomo, Diana Andrianita Kusumaningrum, Tatan Kostaman, Zultinur Muttaqin, Aqdi Faturahman Arrazy

**Affiliations:** 1 Research Center for Animal Husbandry, Research Organization for Agriculture and Food, National Research and Innovation Agency (BRIN), Bogor, Indonesia; 2 Division of Veterinary Reproduction, Faculty of Veterinary Medicine, Universitas Airlangga, Surabaya, Indonesia

**Keywords:** Molecular weight, protein, SDS-PAGE, semen, seminal plasma, testosterone

## Abstract

**Objective:**

This study aims to determine the protein profile based on molecular weight (MW) and testosterone levels in seminal plasma (SP) that correlates to the semen quality of candidate Madura bulls.

**Material and Methods:**

A total of 10 male candidate madura bulls underwent semen evaluation (motility, viability, membrane plasma integrity (MPI), and sperm concentration). The centrifuge was run at 1,200 rpm (4°C) for 20 min to collect SP. SP testosterone levels were measured using an Enzyme-linked immunosorbent assay. The characterization of SP proteins in Madura bulls was done using 1D sodium dodecyl-sulfate-polyacrylamide gel electrophoresis (SDS-PAGE) analysis. All parameters were analyzed using Pearson correlation analysis.

**Result:**

The results of the SDS-PAGE analysis found eight protein bands with the highest MW of 110 kDa and the lowest of 12 kDa. The mean and SD of SP testosterone levels were 20.58 ± 8.56 ng/ml, motility 59.32% ± 20.14%, viability 67.45% ± 20.22%, MPI 32.77% ± 16.52%, and sperm concentration 1,002.64 ± 429.33 10^6^/mm^3^. Proteins with MWs of 110 and 91 kDa significantly correlated with MPI, and 110 kDa negatively correlated with sperm concentration (*p* < 0.05). Proteins with MWs of 73 and 36 kDa significantly correlated with SP testosterone levels, while proteins with MWs of 29 kDa significantly correlated with sperm viability (*p* < 0.05).

**Conclusion:**

The expressed protein fraction based on MW is closely related to the quality of semen, so it has the potential to be a biomarker of semen quality. Further research is needed to determine the specific proteins in certain fractions.

## Introduction 

The government has made various efforts to increase the population of beef cattle in Indonesia. Reproductive efficiency is an important aspect of increasing the beef cattle population by distributing frozen semen from superior bulls through an artificial insemination (AI) program [1]. In this decade, farmers have tended to prefer crossbreeding local cattle with frozen semen from European bulls such as Simentals and limousines [2]. Crossbred cattle tend to produce higher levels of meat and are more expensive in the market than local beef cattle. Crossbred cattle are economically more efficient because they have higher birth weights, faster weaning to adulthood, faster sexual maturity, and higher milk production than purebred cattle [3]. Madrasin cattle (Madura × Limousine) have a higher chest girth, whiter height, and longer body length than Madura cattle [4]. Economically, cross-breeding cattle is beneficial for breeders and the country. However, local cattle will become extinct if the cross-breeding of local and exotic cattle continues.

Madura cattle are indigenous beef cattle whose germplasm needs to be maintained for their existence. However, the quality standards for Madura cattle still need further research to improve genetic quality and secure local beef cattle genetic resources [5]. Sumenep was one of the regencies in Indonesia that launched an AI village to increase Indonesia’s local beef cattle population [6]. The success of AI cannot be separated from the fertility aspect, which is influenced by the semen quality of the selected bulls [7]. Fertilization is the process of sperm fertilizing the egg and maintaining embryonic development during gestation [8]. In addition, bull fertility is an accurate prediction to determine superior male genetic selection [1], which is determined by the number of livestock born from the sperm of a particular bull [9]. The selection of the bulls used for semen production was carried out based on the evaluation of the phenotype and characteristics of the semen [10]. The evaluation for selecting superior bull candidate cattle was carried out by observing reproductive quality through the evaluation of motility, concentration, morphology, and intact acrosome caps of sperm [11]. Meanwhile, those methods had not been 100% successful with the results of the conception rate in the field, which is only 20%–40% [10]. Routine macroscopic and microscopic evaluation of semen quality is not sufficiently accurate in determining bull fertility [7].

Specific biochemical elements in the liquid portion of semen, known as seminal plasma (SP), also had an impact on semen quality. SP is secreted from the testes, epididymis, and mostly accessory glands that contain sperm fertility factors [12]. It had been previously reported that proteins of SP have a role in sperm capacitation and acrosome reactions during the fertilization process, as well as the initiation of embryonic development [13,14]. In several mammals, binder of sperm proteins were found in SP and played a role in sperm capacitation [15]. In addition, protamine 1 was found in the bovine SP, which was associated with the processes of spermiogenesis and sperm maturation in the cow reproductive tract [1]. Based on these findings, a molecular approach is needed to evaluate semen quality for the determination of bull fertility. The protein profile in SP can be detected using the most recent technique, namely proteomics. According to Selvaraju et al. [16], a molecular approach with proteomic techniques could increase the accuracy of semen fertility. This method was able to discover the mechanisms of biological reproduction related to sperm function and their interactions with oocytes [17], and it could detect disturbances in the process of spermatogenesis to fertilization molecularly [18].

The simplest proteomic method to identify protein profiles is to use sodium dodecyl sulfate-polyacrylamide gel electrophoresis (SDS-PAGE). This method has been widely used with the basic principle of separating proteins based on molecular weight (MW). SP proteins of 15 and 22–24 kDa were known to correlate with fertility [19]. Kumar et al. [20] reported that a 22 kDa protein was known as beta nerve growth factor, which is related to the ovulation induction factor. SP proteins were associated with sperm quality. Previous studies had reported that SP proteins found at 35 kDa [10], 45 kDa [21], 58 kDa [17], and 73–74 kDa [22] were correlated with sperm motility. In addition, the protein MW of 40 kDa was associated with protecting sperm against oxidative stress during freezing [23]. This research aims to determine the protein profile based on MW and testosterone levels in SP that correlates to the semen quality of candidate Madura bulls.

## Materials and Methods

### Ethical statement

This research had been approved for ethical clearance by the Animal Welfare Committee of the National Research and Innovation Agency with decree number 080/KE.02/SK/10/2022. The bulls were reared at the TIU Breeding Livestock and Animal Health Unit of Pamekasan Regency, Madura, Indonesia. Semen collection was carried out in the morning by skilled technicians used to handle bulls. There was no use of anesthetics on animals before the collection of semen.

### Animals

A total of 10 Madura cattle candidates for the bulls were used in this study. The criteria for selecting bulls are healthy condition, age ranges of 3–5 years, body weight of 224–330 kg, normal genitalia, and good libido. The feed given was elephant grass (10% of body weight) and concentrate (9 kg) with a crude protein of 16%–17%, and water was given ad libitum. Madura bulls were reared 8 m above sea level at coordinate latitude −7.161367 and longitude 113.482498. The location in the wet season was particularly cloudy and windy in the dry season. The temperature varied between 28°C and 35°C with a humidity of approximately 80%.

The semen of Madura bulls was collected using an artificial vagina in the morning (2 times a week) for 3 weeks. Macroscopic and microscopic evaluation is needed to determine the quality of semen [motility, viability, membrane plasma integrity (MPI), and concentration parameters]. The centrifuge was performed on the semen to separate the sperm and SP at a speed of 1,200 rpm at 4°C (20 min). Samples were obtained and stored at −20°C for further research.

### Evaluation of sperm quality

Semen quality variables, including motility, viability, MPI, and concentration of sperm, were observed in this study. Sperm motility was examined with a 1:1 drop of semen and NaCl placed onto a clean glass object and covered with a glass cover, then observed under a 400× magnification microscope. The assessment of motility was expressed by the individual movement of sperm, marked with the percentage of moving forward sperm (progressive motility) to the total sperm. Assessment of sperm viability was based on a comparison of live sperm numbers (colorless heads) with the total sperm observed and expressed in percent (%). Eosin-negrosin staining was used to determine sperm viability with a ratio of 3:1 (Eosin-Negrosin and semen). The mixture was then smeared on a glass object and placed 2–3 cm from the Bunsen to fixate, then observed under a 400× magnification microscope.

MPI was done using a hypoosmotic swelling test with a ratio of 1:10 of semen and a mixed solution of fructose and NaCl. Incubate the sample in the water bath at a temperature of 37°C for 30 min. The sample needed to be stained with Eosin-Negrosin to facilitate the assessment and then observed using a 400× magnification microscope in ten different fields of view. A sperm with an MPI will display a coiled tail and be colorless, whereas sperm with an incomplete MPI will display a straight tail and be red.

A spectrophotometer (Photometer SDM 1 Minitube) was used to measure the sperm concentration with a 535 nm wavelength that had been calibrated for bovine. The spectrophotometer was set to 0 using water saline, then as much as 20 μl of semen was mixed in 2 ml of 0.9% physiological NaCl, and the concentration was measured [24].

### SP testosterone levels analysis

SP testosterone levels were measured using the Testosterone Enzyme-linked immunosorbent assay kit (BT-Lab, Shanghai, China). About 40 μl of testosterone antigen and 10 μl of the sample were added and mixed into the well plate, which was then incubated for 60 min at room temperature. After the incubation, wash the plate with a buffer solution for 30–60 sec, five times each, then dry using an absorbent tissue. Each well was added with 50 μl horseradish peroxidase and incubated for 30 min at room temperature, then washed the plate five times. Each well was filled with 50 μl of A substrate solution, then 50 μl of B substrate. After incubation for 10 min, each well was added 50 μl of stop solution. The optical density needed to be determined in each well using a microplate reader that was set to a wavelength of 450 nm to measure testosterone levels [25].

### Identification of SP proteins

The identification of SP protein using 1-D SDS-PAGE analysis is based on the Laemlli method [26]. Before SDS-PAGE analysis, samples were measured for protein concentration using a Nanodrop 2,000 spectrophotometer (Thermo-Scientific) with a volume of 0.2 µl. The concentration of each sample was diluted by adding phosphate-buffered saline to obtain a final concentration of 20 mg/ml. Mix a protein sample with sample buffer (2× Laemmli sample buffer, Bio-Rad) in a ratio of 1:1 and then denature proteins in a dry block at 95°C for 5 min. About 10 μl of protein samples were carefully poured into each well of acrylamide gel that contained 4% stacking gel and 12% separating gel. 8 μl of protein standards marker (Pre-stain standard Thermo-Scientific) was put into one well. After that, the electrophoresis was run at 80 volts for 2–3 h until all the proteins and ladder were down. Acrylamide gel was placed in a box, and then the staining solution of Coomassie blue R-250 was poured over it until it was covered well. It was incubated for 30 min with a shaker, then washed with a destaining solution overnight using a shaker.

### Statistical analysis

**Figure 1.**
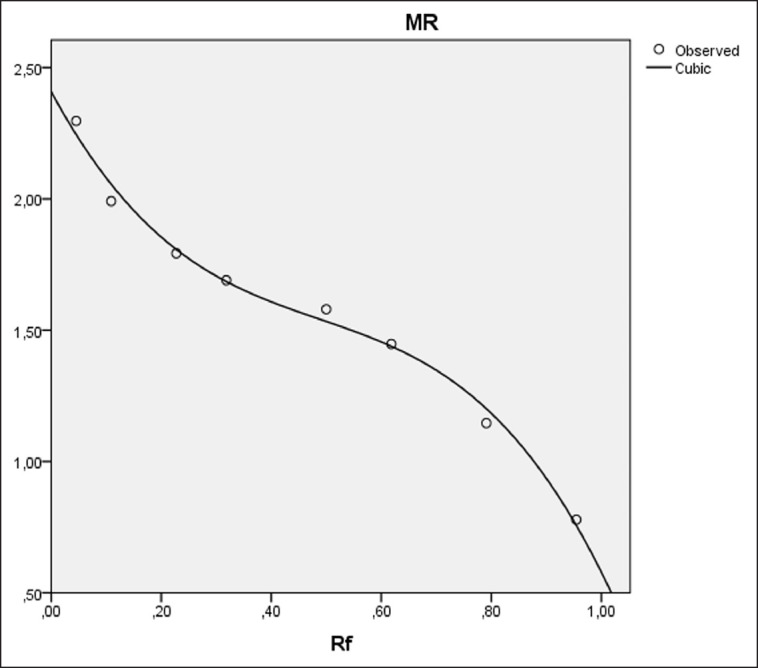
Regression curve of protein standard markers with the equation of logarithm *Y* = 3.895 (*X*) + 6.509 (*X *^2^) − 4.445 (*X *^3^) + 2.409.

Parameters of SP testosterone levels and semen quality were analyzed descriptively, and the relationship between variables was analyzed using Pearson's correlation coefficient. The protein bands from the acrylamide gel were measured to obtain MW based on the regression equation of the protein standard marker, namely the logarithm *Y* = 3.895 (*X*) + 6.509 (*X*2) − 4.445 (*X*3) + 2.409 (Figure 1). In this case, *Y* is the logarithm of the MW protein of Madura bulls, while *X* is the retention factor value or the result of the division of the distance of the protein band movement from the starting point by the distance of the tracking color movement. The final MW results are obtained by converting the logarithm *Y* to the antilogarithm of *Y*. The correlation between each MW, testosterone levels, and semen quality uses the Spearman correlation coefficient. All data were analyzed using Statistical Package for the Social Sciences version 25.

## Results and Discussion

In this study, the semen quality of Madura bulls varied among individuals, with average motility of 59.32% ± 20.14%, viability of 67.45% ± 20.22%, MPI of 32.77% ± 16.52%, and sperm concentration of 1,002.64 ± 429.33 10^6^/mm^3^ (Table 1). In general, the results of the semen quality of Madura bulls were not much different from the previous studies conducted by Kurniawan et al. [27] in the same age ranges. However, the semen quality of Madura bulls was relatively low and did not meet the standard for frozen semen production in bulls. According to INSA 4869-1:2017 [28], the standard of frozen semen quality must have a minimum motility of 40%. The motility of the raw semen of Madura bulls in this study was 59.32%. For further processing, frozen semen had to have at least 60% of the motility of raw semen.

The determination of semen quality in Madura bull candidates can also be evaluated with testosterone levels. SP testosterone levels of Madura bulls were 20.58 ± 8.56 ng/ml (Table 1). These results were higher compared to the previous studies in cross-breed bulls (FHxTharparkar) at 2.13 ± 1.04 ng/ml [29] and Bali bulls at 4.87 ± 1.27 ng/ml [30]. Different species of bulls might cause differences in testosterone levels because they are influenced by genetic factors. Genetic associations in each individual and species were used for the genotype selection of bulls that have high testosterone levels [31]. Meanwhile, SP testosterone levels in Madura bulls were lower than serum testosterone levels by 23.34 ± 9.91 ng/ml [32]. The hormone testosterone is produced by Leydig cells from the seminiferous tubules, while SP is mostly secreted by the accessory glands and only a small amount by the seminiferous tubules. High levels of the hormone testosterone in the blood serum might be produced by the secretion of a process of seminiferous tubule reabsorption and passing through the hematotesticular barrier, in which each steroid hormone has a different permeability [33]. Most of the SP fluid was produced from the secretion of the accessory glands, and only a small part was produced from the seminiferous tubules, causing testosterone levels in SP to be lower than in blood serum [34]. Hafizuddin et al. [35] reported that testosterone levels are correlated with semen quality. In this study, SP testosterone levels did not correlate with the variables of semen quality in Madura bulls (*p* > 0.05). The low levels of SP testosterone might not significantly affect semen qualities in Madura bulls. These results were presumably caused by spermatogenesis failure or endocrine resistance conditions [36], where spermatogenesis directly correlated with sperm motility and concentration [37].

Variables among semen quality showed a positive correlation, where sperm motility was strongly correlated with viability and MPI (*p* < 0.05), and sperm viability was strongly correlated with MPI (*p* < 0.05) (Table 2). Sperm motility was affected by the number of live sperm. The high sperm motility is due to the high number of living sperm in SP. Sperm motility and viability could decrease due to pressure on sperm during the freezing process, causing changes in MPI quality [38]. MPI quality was very important in maintaining sperm function because sperm that have an intact plasma membrane can survive the process of transmigration from the bull to the cow reproductive tract and also the process of fertilization [39].

**Table 1.** SP testosterone levels and the semen quality of Madura bulls.

Parameter	Min	Max	Mean	SD
SP testosteron (ng/ml)	3.25	31.29	20.58	8.56
Motility (%)	20.00	90.00	59.32	20.14
Viability (%)	25.00	96.00	67.45	20.22
MPI (%)	8.00	68.00	32.77	16.52
Sperm concentration (10^6^/mm^3^)	361.00	1,892.00	1,002.64	429.33

Min: minimum; Max: Maximum; SD: Standard Deviation; and MPI: Membrane plasma integrity.

**Table 2.** Coefficient correlation (*r*) among variables (SP testosterone levels, motility, viability, MPI, and concentration of sperm).

Variable	Motility	Viability	MPI	Sperm concentration
SP testosteron	0.204	0.233	0.356	0.302
Motility		0.966*	0.785*	0.363
Viability			0.813*	0.364
MPI				0.276

*Significant correlation (*p* < 0.05).

**Figure 2.**
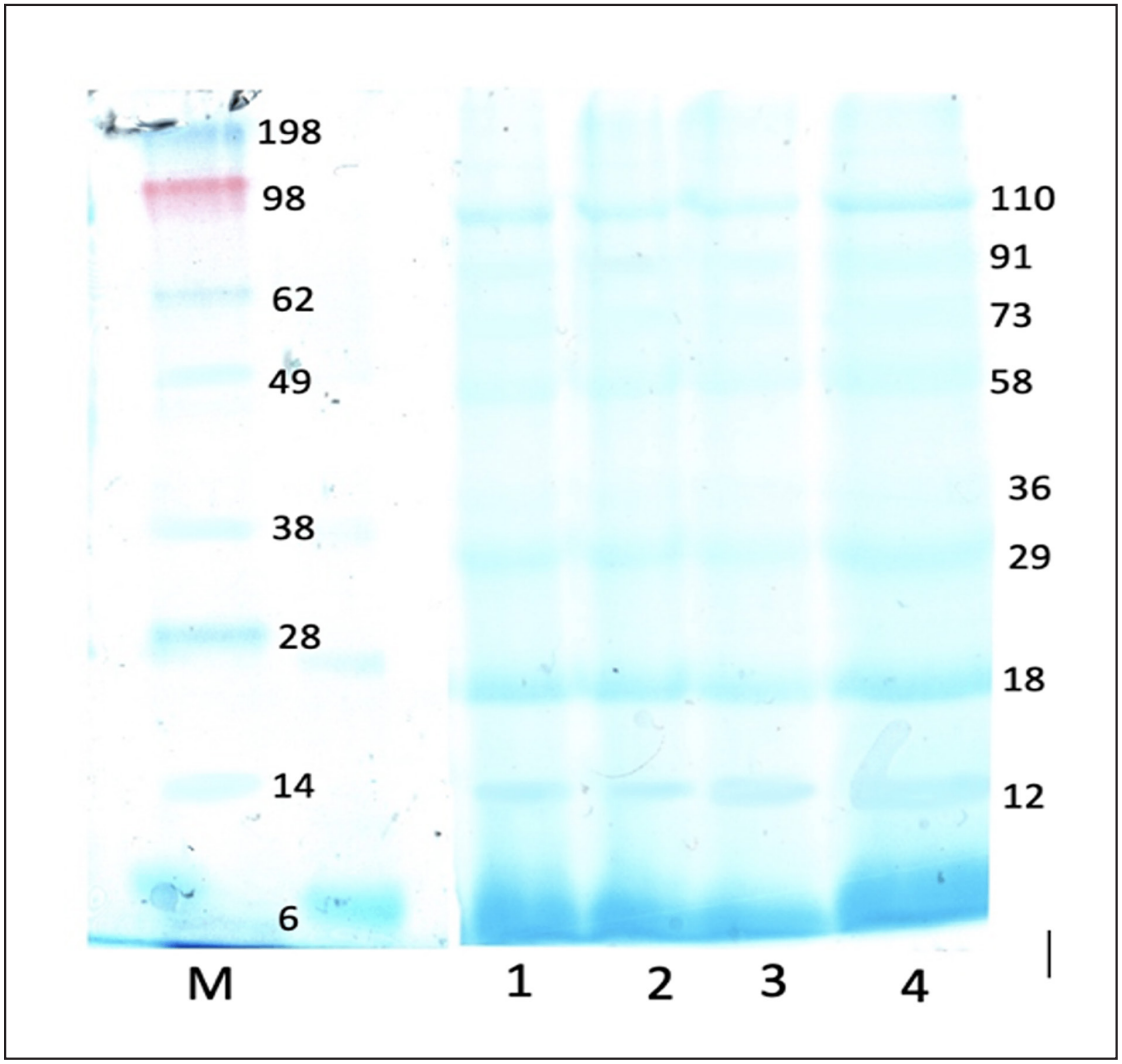
Protein fractions and MW (kDa) of SP Madura bulls using SDS-PAGE analysis.

Semen quality is also determined based on specific proteins present in SP. In this study, there were 8 protein fractions expressed in the SP of Madura bulls, with the highest MW of 110 kDa and the lowest MW of 12 kDa (Figure 2). The number of protein fractions expressed in SP was less than in previous studies in Simmental bulls, which found 10 protein fractions (15–181 kDa) [11] and in Crossbreed Jerseys, which found 13 protein fractions (8.5–204 kDa) [40]. Different species might cause the number of protein fractions and MWs expressed in SP to vary. Semen quality and the success of fertilization in each individual and species depend on reproductive cell function that is associated with spermatogenesis, capacitation (epididymal maturation and capacitation), and the acrosome reaction during fertilization. Meanwhile, protein molecules associated with sperm can affect the structure and metabolism, which are different for each individual [41]. SP contained many proteins, and each protein had association and dissociation relationships at specific peptide conditions based on a certain MW [42].

The correlation between the MW protein in SP and semen quality is presented in Table 3. The expression of SP protein fractions showed a positive and negative correlation in each variable of the semen quality of Madura bulls. MW proteins of 110 and 91 kDa were significantly correlated with MPI (*p* < 0.05) with strong correlation coefficients of 0.987 and 0.964, respectively. The MW of 110 kDa was negatively correlated with sperm concentration, with a correlation coefficient of −0.965 (*p* < 0.05). Proteins of 73 and 36 kDa were significantly correlated with SP testosterone levels, and 29 kDa was significantly correlated with sperm viability (*p* < 0.05). The correlation between proteins of 73 and 36 kDa and SP testosterone levels had a strong correlation coefficient of 0.981 and 0.988, respectively. Meanwhile, the protein of 29 kDa had a strong correlation coefficient on sperm viability of 0.995. Sperm motility was generally correlated with SP protein but did not show a significant correlation (*p* > 0.05). A previous study reported that SP proteins are correlated with sperm fertility [11]. The high content of SP protein will result in high progressive motility, viability, and MPI of sperm [43]. However, not all SP proteins are expressed in highly fertile bulls. Pardede et al. [39] reported that several dominant proteins, such as spermadhesin 2 and clusterin, are also found in many low-fertile bulls.

Proteins with MWs of 110 and 91 kDa were thought to be A-kinase anchoring proteins (AKAPs) [44], which are strongly correlated with MPI. AKAP proteins were able to form multiprotein complexes in biochemical processes to regulate flagella and cilia regulation in sperm so that they could affect sperm motility [45]. AKAPs also play roles in motility, capacitation, and acrosome reactions during the fertilization process [46]. The role of these proteins was thought to be related to MPI in protecting sperm from an imbalance of osmotic pressure [47]. A protein of 110 kDa had a negative correlation with the concentration of sperm. These results were in line with previous studies, which reported that AKAP9 interfered with fertility and somatic germ cell organization in the process of spermatogenesis [48]. Sperm concentration in bovines was affected by the process of spermatogenesis, which occurred for 60 days from the initial formation of primary spermatocytes in the germ cells until the ejaculated sperm [49]. The presence of disruption in the spermatogenesis process could cause a decrease in sperm concentration.

**Table 3.** Coefficient correlation between SP proteins based on MW and sperm quality.

SP protein (kDa)	Motility	Viability	MPI	Sperm concentration	SP testosterone
110	0.642	0.765	0.987*	−0.965*	0.894
91	0.302	0.805	0.964*	−0.794	0.677
73	0.861	0.283	0.733	−0.868	0.981*
58	0.314	0.313	0.860	−0.686	0.785
36	0.739	0.462	0.898	−0.926	0.988*
29	0.471	0.995*	0.765	−0.778	0.523
18	0	0	0	0	0
12	0	0	0	0	0

* Significant correlation (*p* < 0.05).

Proteins with MWs of 73 and 36 kDa might be involved in the regulation of steroidogenesis because they correlate with SP testosterone levels. Fraser et al. [50] found that the protein fraction >40 kDa of the anelin signaling pathway, which consists of guanine nucleotide-binding protein subunit alpha-13 (GNA13), myocyte enhancer factor 2D (MEF2D), and sphingosine kinase 2 (SPHK2), plays a role in the process of steroidogenesis. These three proteins are involved in the anelin signaling pathway, which is detected in SP and contributes to sperm function. Anelin signaling pathways were reported to play a role in the regulation of steroidogenesis, proliferation, apoptosis, and inhibition of kinase signaling pathways [51]. The GNA13 protein was a G protein subunit alpha that was expressed in the cytoplasm of Leydig cells, seminiferous tubular epithelial cells, and cells differentiating from elongated spermatids to mature sperm so that it is possible to be related to the processes of steroidogenesis and spermatogenesis [52]. The role of the other two proteins (MEF2D and SPHK2) in the process of steroidogenesis is still unclear. Involved in the anelin signaling pathway, it is possible that MEF2D and SPHK2 played a role together with the GNA13 protein in the regulation of steroid hormones.

Proteins with a MW of 29 kDa are correlated with the viability of sperm. Matrix metalloproteinase was found in sufficient numbers in the >40 and <40 kDa protein fractions, which play a role in protecting membrane integrity and sperm viability [50]. Clusterin protein was also found in abundance in SP as an inhibitor of oxidative damage and lysis of sperm so that it could maintain sperm viability [53]. Other proteins, such as glutathione peroxidase, are reported to increase the antioxidant effect, which can maintain the life of sperm [54]. SP proteins were varied and related to viability at a MW of 29 kDa and need further study.

The determination of SP protein based on MW and SP testosterone levels as biomarkers of semen quality in Madura bulls can be used as a reference for the selection of superior bulls. However, further analysis is needed to correctly and accurately determine protein profiles using a proteomic approach. The protein based on MWs can be further analyzed using nano-liquid chromatography-mass spectrometry (LC/MS) to identify protein profiles that have potential as biomarkers of the bull's semen quality. In addition, it is also necessary to measure testosterone hormones in the blood serum because the levels are higher and more accurate to determine semen quality.

## Conclusion

In general, semen quality in Madura bulls is still quite good but does not meet qualified standards for frozen semen production. The semen quality of Madura bulls correlated with each other among variables, but it did not correlate with SP testosterone level. The protein fraction expressed in SP correlates with several sperm qualities that have the potential to be biomarkers of semen quality. The determination of SP protein profiles based on MW can be used as a reference, but it is still not enough to accurately determine the protein profile because the MWs are almost the same and have different charges and affinities. Further research needs to be conducted through a proteomic analysis approach using the nano-LC/MS method so that specific and accurate protein types can be obtained.

## List of Abbreviations

ELISA, Enzyme-linked immunosorbent assay; HRP, Horseradish peroxidase; MPI, Membrane plasma integrity; MW, Molecular weight; nano–LC/MS, nano liquid chromatography-mass spectrometry; SDS, Sodium dodecyl-sulfate; SP, seminal plasma; TIU, Technical implementation unit; TEMED, Tetramethylethylenediamine.
